# High endemicity of *Opisthorchis viverrini* infection among people in northern Cambodia confirmed by adult worm expulsion

**DOI:** 10.1038/s41598-023-36544-z

**Published:** 2023-06-14

**Authors:** Bong-Kwang Jung, Sooji Hong, Taehee Chang, Jaeeun Cho, Seungwan Ryoo, Keon Hoon Lee, Jeonggyu Lee, Woon-Mok Sohn, Sung-Jong Hong, Virak Khieu, Rekol Huy, Jong-Yil Chai

**Affiliations:** 1 MediCheck Research Institute, Korea Association of Health Promotion, Seoul, 07649 Republic of Korea; 2 Department of Parasitology and Tropical Medicine, and Institute of Health Sciences, Gyeongsang National University School of Medicine, Jinju, 52727 Republic of Korea; 3 Convergence Research Center for Insect Vectors, Incheon National University, Incheon, 22012 Republic of Korea; 4 National Center for Parasitology, Entomology and Malaria Control, Ministry of Health, Phnom Penh, Cambodia; 5 Department of Tropical Medicine and Parasitology, Seoul National University College of Medicine, Seoul, 03080 Republic of Korea

**Keywords:** Microbiology, Diseases, Medical research

## Abstract

*Opisthorchis viverrini* infection is an emerging disease in Cambodia, especially in central and southeastern areas. However, its status in northern areas bordering Lao PDR has been relatively unknown. The present study was performed to investigate the status of *O. viverrini* infection among people in Preah Vihear and Stung Treng provinces through fecal examination to detect eggs and recovery of adult flukes from some of the egg-positive cases. Fecal examinations were performed on a total of 1101 people from 10 villages in the 2 provinces using the Kato-Katz thick smear technique. For recovery of adult flukes and other helminth parasites 10 volunteers positive for eggs of *Opisthorchis viverrini* and/or minute intestinal flukes (Ov/MIF), in Kampong Sangkae village, Preah Vihear province, were administered a single oral dose of 40 mg/kg praziquantel plus 5–10 mg/kg of pyrantel pamoate and purged with 40–50 g magnesium salts. Adult trematodes, together with nematodes and cestodes expelled in diarrheic stools were collected under a stereomicroscope or with the naked eye. The proportion of egg-positive cases for overall liver and intestinal helminths was high but not notably different between the 2 provinces, 65.5% in Preah Vihear versus 64.7% in Stung Treng. The average proportion of Ov/MIF egg-positive cases was 59.8%. A total of 315 adult specimens of *O. viverrini* were recovered from the 10 volunteers (4–98 specimens per individual; mean, 32). A smaller number of *Haplorchis taichui* adults, an intestinal fluke, were found mixed-infected in 7 (103 specimens in total; 1–31 per individual; mean, 15) of the 10 volunteers. Adult specimens of hookworms, *Enterobius vermicularis, Trichostrongylus* sp., and a *Taenia* tapeworm strobila were recovered in some cases. Based on the results, it has been confirmed that the surveyed areas in Preah Vihear and Stung Treng provinces, Cambodia, are highly endemic areas of *O. viverrini* infection with a low-grade mixed infection with *H. taichui*.

World Health Organization (WHO) has recognized foodborne trematodiases, including liver, lung, and intestinal fluke infections, as an essential group of neglected tropical diseases^1^. Among the liver flukes, *Opisthorchis viverrini, Opisthorchis felineus*, and *Clonorchis sinensis* are the 3 major species causing liver diseases in humans and animals. For completion of their life cycles, they need freshwater snail species, for example, *Bithynia* spp. for *O. viverrini* and *O. felineus*, and *Parafossarulus* spp. for *C. sinensis*, to develop into cercariae, freshwater fish species, including cyprinoid fish, to develop into metacercariae, and mammalian hosts, including humans and domestic animals, to develop into adult flukes in the bile duct^2^. Consumption of raw or undercooked freshwater fish is the principal mode of infection^2^. The infection is characterized by cholangitis in acute stages and liver cirrhosis and cholangiocarcinoma (more commonly in *O. viverrini* and *C. sinensis*) in chronic stages^2^. Common clinical manifestations include fever, jaundice, anorexia, weight loss, fatigue, yellow sclera, and decreased liver functions^2–4^. *O. viverrini* is the responsible species in the Greater Mekong Subregion, especially in Thailand, Lao PDR, and southern parts of Vietnam^3^. *O. felineus* is distributed mainly in Eastern Europe, and *C. sinensis* is distributed in far East Asia (China and Korea) and northern parts of Vietnam^2^.

In Cambodia, information on the status of *O. viverrini* infection has historically been scarce. Recently, however, *O. viverrini* infection became acknowledged as an emerging disease based on published as well as unpublished observations^4^. In 2006, an *O. viverrini* initiative was launched as the National Helminth Control Program^4^. However, this program was mostly focused on the detection of endemic areas of *O. viverrini* through nationwide fecal examinations using the Kato-Katz fecal smear technique^4^. The results revealed that the geographical distribution of *O. viverrini* in Cambodia seemed to be almost countrywide, i.e., in 22 of the total 24 provinces surveyed; the prevalence in egg positive rate of *O. viverrini* and/or minute intestinal flukes (Ov/MIF) ranged from 0.1 to 47.5% depending on different localities^4,5^. The highest prevalence was found in central and southeastern provinces, including Takeo (23.8–47.5%), Kampong Thom (34.8%), Kampong Cham (24.0%), and Kandal (20.2%)^4–7^.

Regarding the two northernmost provinces bordering Lao PDR, Preah Vihear and Stung Treng, the prevalence was reported to be relatively low, 2.7 and 2.5%, respectively, based on a nationwide survey^5^. However, according to other reports, the prevalence in these areas was variable; for example, 1.4% in 20 villages (2010)^4^, 5.1% in 1 village (2014)^8^, and 45.7% in 1 village (2010)^4^ in Preah Vihear, and 0% in 11 villages (2000 and 2007)^4^ and 2.3% in 4 villages (1998)^9^ in Stung Treng. Thus, it is considered that the endemicity of *O. viverrini* and/or MIF in Preah Vihear and Stung Treng provinces varies depending on the locality (riversides or remote from the river) and target population (children or adults) and should be further investigated in different areas, particularly among people living along the rivers and streams.

In addition, *O. viverrini* and MIF produce visually similar eggs^10^, although they are biologically dissimilar organisms and parasitize different organs in the definitive host. Hence, parasitological diagnostic tests have imperfect specificity when there are co-infections with *O. viverrini* and MIF, and thus these eggs are expressed as Ov/MIF eggs. It is needed to confirm whether the Ov/MIF eggs detected in the present study are really those of *O. viverrini* or of MIF, or mixed-infected. For this, adult worm recovery from egg-positive individuals after chemotherapy is one of the procedures that can be applied^11,12^. Sequencing of relevant genes, for example, 18S and 28S rDNA, from the eggs can be an alternative method showing high sensitivity and high specificity^13^.

In the present study, we surveyed the infection status of liver and intestinal helminths among riparian people in 10 villages of Preah Vihear and Stung Treng provinces (Fig. 1) bordering Lao PDR using the Kato-Katz technique to detect fecal eggs. In addition, 10 volunteers from Preah Vihear province positive for Ov/MIF eggs were prescribed praziquantel and pyrantel pamoate followed by purging with magnesium salts, and the adult flukes, nematodes, and cestodes expelled in diarrheic stools were collected and morphologically identified.

**Figure 1:**
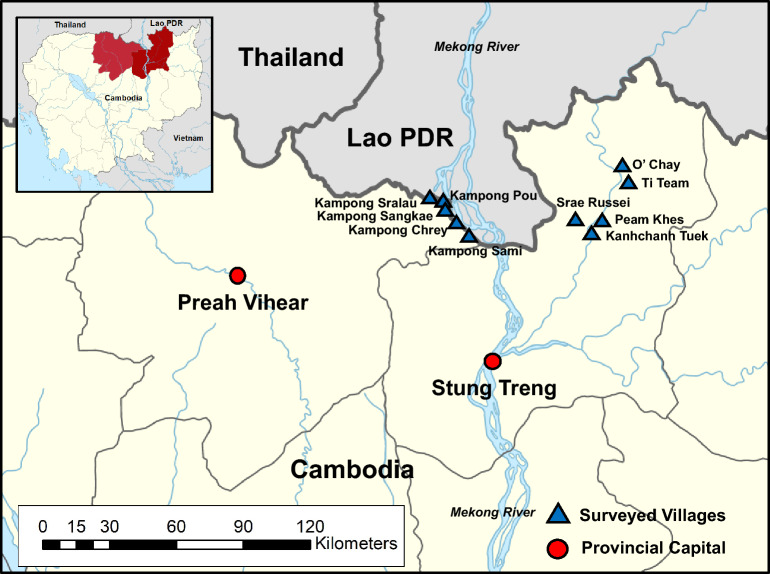
Map showing the surveyed areas of Preah Vihear and Stung Treng province, Cambodia. Ten villages (small blue triangles) located along the tributaries of the Mekong River were included in this study.

## Results

### Overall prevalence and intensity of infection of Ov/MIF and other helminthes

The proportion of overall helminth egg-positive cases was very high, 64.9%, with a 95% confidence interval (CI) of 62.1–67.7%, among the people surveyed in 5 villages each of Preah Vihear and Stung Treng provinces (Table 1). The eggs of Ov/MIF were the most commonly identified species with an average prevalence of 59.8% (56.8–62.6%), which slightly varied among different villages and between the two provinces, 58.3% (52.7–63.7%) in Preah Vihear and 60.3% (56.9–63.7%) in Stung Treng (Table 1). The arithmetic mean EPG (number of eggs per gram of feces) of Ov/MIF was 862 per egg-positive person (n = 658). The highest prevalence was found in Kampong Sangkae village (80.9%) in Preah Vihear province. The second highly prevalent helminth was hookworms (12.2%; 10.1–13.9%; mean EPG 413), followed by *Taenia* spp. (2.4%), *Trichuris trichiura* (1.5%; 0.9–2.4%; mean EPG 67), *Schistosoma mekongi* (1.0%), *Enterobius vermicularis* (0.8%), echinostomes (0.6%), *Trichostrongylus* sp. (0.4%), and others (0.5%). Others included dicrocoeliid eggs and *Hymenolepis diminuta* eggs. The species of echinostomes was difficult to determine by the eggs, although it was highly suggested to be *Echinostoma mekongi*. However, the eggs of *Ascaris lumbricoides* were not detected in both surveyed provinces.

**Table 1:** Prevalence of helminth eggs by the Kato-Katz technique among people in Preah Vihear and Stung Treng province, Cambodia in 2018.

Target area		No. of exam	No. of overall helminth egg posit. cases (%)	No. of overall helminth egg positive cases (%)								
Province	Village			Ov/MIF	Hw	*Taenia* spp.	Tt	Sch	Ev	Ech	Tricho	Others
Preah Vihear	Kampong Chrey	42	28 (66.2)	19 (45.2)	10 (23.8)	2 (4.8)	1 (2.4)	3 (7.1)	0 (0.0)	0 (0.0)\	2 (4.8)	0 (0.0)
	Kampong Sangkae	89	73 (82.0)	72 (80.9)	20 (22.5)	3 (3.4)	1 (1.1)	0 (0.0)	1 (1.1)	0 (0.0)	1 (1.1)	2 (2.2)
	Kampong Sralau	74	30 (40.5)	23 (31.1)	8 (10.8)	0 (0.0)	2 (2.7)	0 (0.0)	2 (2.7)	0 (0.0)	1 (1.4)	0 (0.0)
	Kampong Sami	50	40 (80.0)	36 (72.0)	3 (6.0)	1 (2.0)	0 (0.0)	3 (6.0)	0 (0.0)	0 (0.0)	0 (0.0)	0 (0.0)
	Kampong Pou	52	30 (57.7)	29 (55.8)	2 (3.8)	0 (0.0)	0 (0.0)	1 (1.9)	0 (0.0)	0 (0.0)	0 (0.0)	0 (0.0)
	Subtotal	307	201 (65.5)	179 (58.3)	43 (14.0)	6 (2.0)	4 (1.3)	7 (2.3)	3 (1.0)	0 (0.0)	4 (1.3)	2 (0.7)
Stung Treng	Kanhchanh Tuek	114	71 (62.3)	66 (57.9)	5 (4.4)	4 (3.5)	2 (1.8)	1 (0.9)	0 (0.0)	1 (0.9)	0 (0.0)	1 (0.9)
	Ti Team	93	59 (63.4)	56 (60.2)	18 (19.4)	0 (0.0)	6 (6.5)	0 (0.0)	0 (0.0)	0 (0.0)	0 (0.0)	0 (0.0)
	Srae Russei	204	143 (70.1)	138 (67.6)	25 (12.3)	4 (2.0)	2 (1.0)	1 (0.5)	1 (0.5)	2 (1.0)	0 (0.0)	0 (0.0)
	Peam Khes	261	158 (60.5)	147 (56.3)	23 (8.8)	6 (2.3)	1 (0.4)	1 (0.4)	4 (1.5)	0 (0.0)	0 (0.0)	2 (0.8)
	O’ Chay	122	83 (68.0)	72 (59.0)	20 (16.4)	6 (4.9)	1 (0.8)	1 (0.8)	1 (0.8)	4 (3.3)	0 (0.0)	0 (0.0)
	Subtotal	794	514 (64.7)	479 (60.3)	91 (11.5)	20 (2.5)	12 (1.5)	4 (0.5)	6 (0.8)	7 (0.9)	0 (0.0)	3 (0.4)
Total		1101	715 (64.9)	658 (59.8)	134 (12.2)	26 (2.4)	16 (1.5)	11 (1.0)	9 (0.8)	7 (0.6)	4 (0.4)	5 (0.5)

*Ov/MIF: *Opisthorchis viverrini*/minute intestinal fluke; Hw: hookworms; Tt: *Trichuris trichiura*; Sch: *Schistosoma mekongi*; Ev: *Enterobius vermicularis*; Ech: echinostomes; Tricho: *Trichostrongylus* sp.; Others: dicrocoeliids and *Hymenolepis diminuta.*

### Age- and sex-specific prevalence and EPG of Ov/MIF eggs

**Figure 2:**
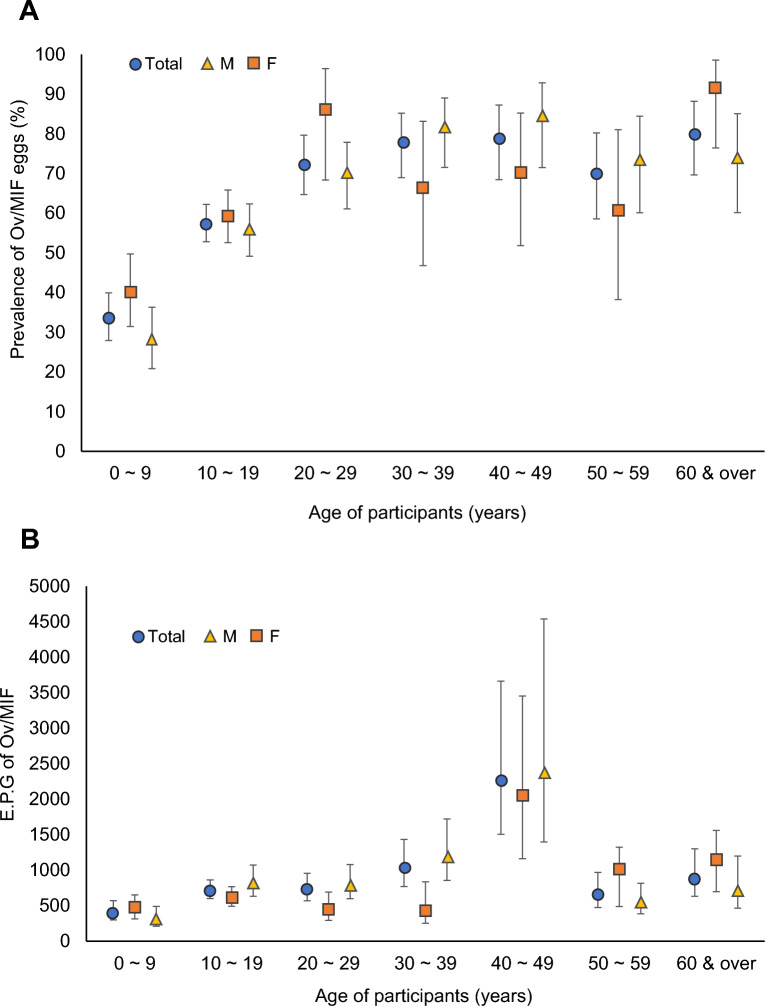
Age- and sex-specific prevalence (mean and 95% confidence interval) (**A**) and EPG (**B**) of Ov/MIF eggs among the riverside people (n = 1101) in Preah Vihear and Stung Treng provinces, Cambodia in 2018. A prominent age tendency is recognizable, but no significant difference is seen between males (n = 432) and females (n = 669).

The prevalence and intensity of infection in EPG of Ov/MIF eggs showed a trend by age (Fig. 2A, B). The average prevalence and EPG (both males and females) were the lowest in the age group 0–9 years, 33.8% (27.9–39.9%) and 408 (300–572), followed by the age groups 10–19 years, 57.5% (52.8–62.2%) and 717 (602–863), 20–29 years, 72.6% (64.7–79.6%) and 730 (570–956), and 30–39 years, 77.8% (68.9–85.2%) and 1034 (770–1433), respectively. Then, the prevalence became steadily high showing a plateau, 78.9–80.0%, in the age groups between 40 and 49 and ≥ 60 years (Fig. 2A). Meanwhile, the mean EPG showed the highest value at 40–49 years, 2273 (1505–3664), followed by a reduction in the ages 50–59 and ≥ 60 years groups (Fig. 2B). The age-prevalence and intensity of infection patterns were almost similar in males and females (Fig. 2A, B) and in Preah Vihear and Stung Treng provinces (data not shown). The average prevalence and EPG were not significantly different between males (59.0%; 54.3–63.6% and 747; 629–896, respectively) and females (60.2%; 56.5–63.9% and 934; 811–1082, respectively). The overall total prevalence and EPG were 59.8% (56.8–62.6%) and 862 (773–965), respectively.

### Adult flukes and other helminths recovered from volunteers in Preah Vihear province

**Figure 3:**
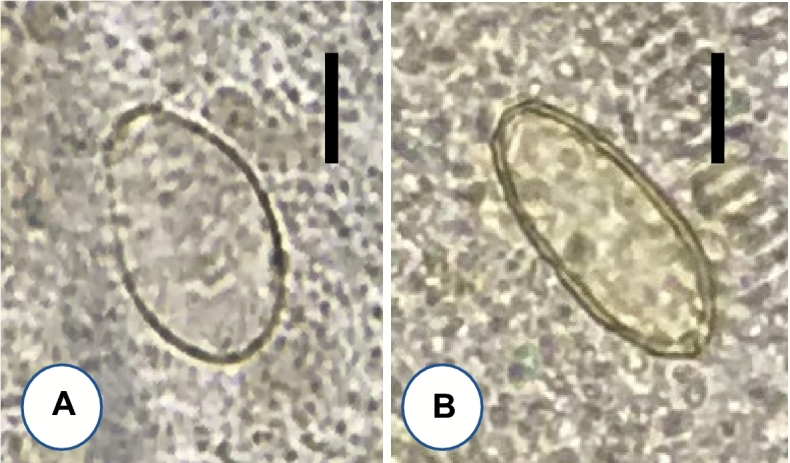
Ov/MIF eggs detected from riparian people in Preah Vihear province, Cambodia. (**A**) Presumed to be an egg of *Opisthorchis viverrini.* (**B**) Presumed to be an egg of *Haplorchis taichui*. Scale bars (A, B) = 10 μm.

**Figure 4:**
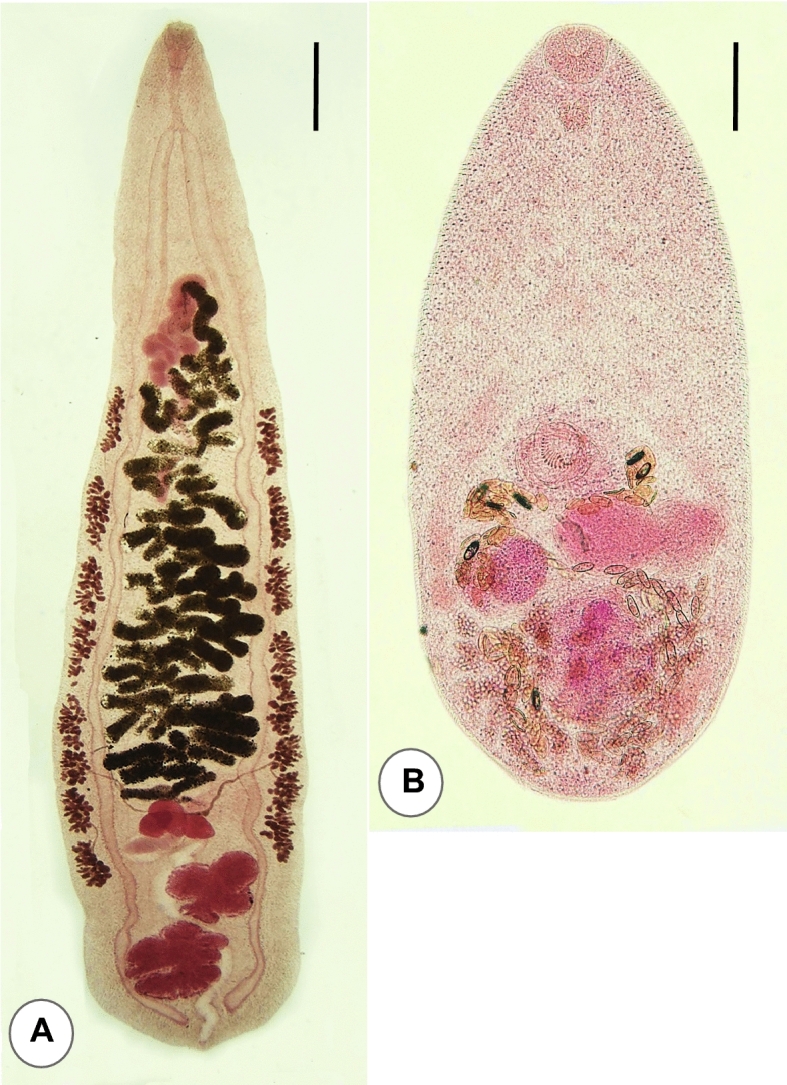
Adult specimens of *Opisthorchis viverrini* (**A**) and *Haplorchis taichui* (**B**) recovered from volunteers in Preah Vihear province, Cambodia. Ventral view. Scale bar; A = 0.75 mm, B = 0.07 mm.

The adult worm recovery was successfully performed in 10 volunteers (5 men and 5 women; aged 21 to 45 years) who were positive for Ov/MIF eggs (Fig. 3), with EPG higher than 240. Eight kinds of helminths, including 2 trematode species, 5 nematode species, and 1 cestode species, were recovered from their diarrheic stools (Table 2). A total of 315 (4–98 per individual) adult specimens of *O. viverrini* were collected from 10 volunteers (Table 2; Fig. 4A), and a total of 103 adult specimens (1–31 per individual) of *H. taichui* (Fig. 4B) were collected from 7 of the 10 volunteers (Table 2). The number of adult worms of *O. viverinii* and *H. taichui* recovered in each case showed a strong positive correlation (*r* = 0.85; *P* = 0.0019) with the EPG (eggs per gram of feces) (Ov/MIF eggs) of each case in the fecal examination (Table 2). EPG was measured by counting the total number of eggs on each Kato-Katz fecal smear, which was multiplied by 24, assuming that the volume of feces on each Kato-Kato smear was 41.7 mg^14^. In addition, 70 adult specimens of hookworms (64 *Necator americanus* and 6 *Ancylostoma ceylanicum*) were collected from 7 volunteers (reported separately by Chang et al.^15^), 57 *Enterobius vermicularis* were from 6 volunteers, 9 *Trichostrongylus* sp. from 3 volunteers*,* 1 strobila of *Taenia saginata* from 1 volunteer (reported separately by Chang et al.^16^), and 1 unknown nematode from 1 volunteer (Table 2).

**Table 2:** Worm recovery after anthelmintic treatment and purging of 10 volunteers from Kampong Sangkae village, Preah, Vihear province, Cambodia in 2018.

Volunteer* code	Age and sex	No. of adult helminth specimens collected						EPG^§^ for Ov/MIF
		*Opisthorchis viverrini*	*Haplorchis taichui*	Hook-worms†	*Enterobius vermicularis*	*Taenia saginata*	Others	
A	38F	98	30	24	8	0	1 (unknown nematode)	13,680
B	45 M	74	0	0	0	0		2808
C	24 M	44	1	11	1	0	3 (*Trichostrongylus* sp.)	2112
D	36F	26	31	2	0	0	2 (*Trichostrongylus* sp.)	336
E	23F	25	29	1	33	0		648
F	26 M	17	7	21	11	0		864
G	21 M	12	0	2	2	0		624
H	27F	8	4	9	2	1‡		480
I	34F	7	0	0	0	0		240
J	26 M	4	1	0	0	0	4 (*Trichostrongylus* sp.)	720
Total		315	103	70	57	1	10	22,512

*Treated orally with 40 mg/kg praziquantel (Shinpoong Pharm. Co., Seoul, Korea) plus 5 mg/kg pyrantel pamoate (Hangzhou.

Minsheng Pharm. Co., Hangzhou, China) and purged with 40–50 g magnesium sulfate.

^†^*Necator americanus* (64 specimens) and *Ancylostoma ceylanicum* (6 specimens)^15^.

^‡^Molecularly confirmed to be *Taenia saginata*^16^.

^§^Eggs per gram of feces; measured by multiplying 24 to the total number of eggs detected on each Kato-Katz fecal smear (amount of feces.

assumed 41.7 mg/smear)^14^.

### Differential morphological features of *O. viverrini* and *H. taichui*

For the speciation of two adult trematode species, their brief morphological features are described here. Adult flukes of *O. viverrini* (Fig. 4A) were elongated and lanceolate, 8.0–10.9 (av. 8.9) mm long and 1.5–2.5 (av. 1.7) mm wide (n = 9). The oral sucker was slightly smaller than the ventral sucker (sucker ratio, 0.9: 1.0). They had 2 obliquely tandem testes, which were 4- (anterior testis) to 5-lobulated (posterior testis) (different from markedly branched testes in *Clonorchis sinensis*), near the posterior end of the body. The vitellaria were distributed laterally from the level of the seminal receptacle or anterior end of the anterior testis up to the 1/3–1/4 length level of the uterus. The vitelline follicles were dense and compact and aggregated into 7–8 groups on each side. The ovary was 5- to 10-lobulated (less lobulated and almost oval in *Opisthorchis felineus*).

Adult flukes of *H. taichui* (Fig. 4B) were small, ovoid to elliptical, 0.73–1.08 (av. 0.88) mm long and 0.38–0.49 (av. 0.42) mm wide (n = 10). They were characterized by the presence of only 1 testis but lacked an expulsor of the seminal vesicle, thus were assigned to the genus *Haplorchis*. They had a small inconspicuous ventral sucker, embedded in the parenchyma, forming a ventrogenital sac with its dorsal and ventral lobes, armed with 12–16 long crescentic and hollow minute spines of 19–30 μm in length. These characters, especially the number and size of minute spines, are differential points from the related species, *Haplorchis pumilio* and *Haplorchis yokogawai*.

## Discussion

The prevalence of Ov/MIF (confirmed to be mostly *O. viverrini*) infection observed in this study appeared to be the highest ever reported in Cambodia. In a nationwide survey performed previously by our group^5^, high prevalences of Ov/MIF were found in Kampong Cham (24.0%) and Takeo province (23.8%), and a relatively lower prevalence was detected in Preah Vihear (2.7%), Stung Treng (2.5%), Kratie (3.4%), and Ratanakiri province (2.8%). In another study by our group^6^, a riparian population in Prey Kabas District in Takeo province revealed a much higher prevalence (47.5%) of Ov/MIF infection. A study by another group^7^ undertaken in 5 provinces of Cambodia reported high prevalences of Ov/MIF infection in Kampong Thom (34.8%) followed by Kampong Cham (33.1%), Takeo (21.4%), and Kandal (18.7%), with no infection in Prey Veng province (0%). Taken together, a total of 6 provinces, including Preah Vihear, Stung Treng, Kampong Cham, Kampong Thom, Takeo, and Kandal, are currently recognized as the major endemic areas of opisthorchiasis in Cambodia. Interestingly, however, in Kratie province, which is located along the mainstream of the Mekong River, and neighbored by the highest endemic areas (Stung Treng, Kampong Thom, and Kampong Cham provinces), the prevalence of Ov/MIF infection was relatively low, 4.6%, among people in 7 riverside villages^17^. Further investigations are needed to properly estimate the prevalence of Ov/MIF infection in Kratie province and other localities of Cambodia along the Great Mekong Subregion.

The high prevalence of *O. viverrini* in the surveyed areas of Preah Vihear and Stung Treng province is comparable with those reported in Lao PDR, the most highly endemic country ever known^18–22^. The reported prevalence of Ov/MIF infection in Lao PDR was 43.8% around Pakse city (Champasak province)^18^, 67.1% in Savannakhet province^19^, 52.0–60.7%^20^ or 81.1%^21^ in Khammouane province, and 53.3% in Vientiane Municipality^22^. Thailand, formerly known as the most highly endemic country for *O. viverrini* infection, became a lower-grade endemic country owing to the active and sustained national control activities over 40 years since the 1980s^3^. The national prevalence in Thailand was 14.7% in 1980–1981, with the highest level of 34.6% in the northeast areas, but the national prevalence decreased to 11.8% in 1996 and 9.6% in 2001^3^. In northeast areas, the prevalence remained at 60.8% in Nakhon Phanom, 38.6% in Sisaket, and 32.6% in Amnat Charoen province by 2009; however, the prevalence in these areas also decreased substantially in 2014 with Nakhon Phanom remained the only province showing the prevalence over 20%^3^. After 2014, however, there were several publications reporting a considerably high prevalence (16.9–28.7%) in northeastern and central Thailand^23–26^. In Vietnam, Phu Yen province and some other mid- to southern provinces have been notified as endemic areas of *O. viverrini*, with the prevalence ranging from 0.3 to 36.9%^3^. A low-grade endemicity was recently reported in lower Myanmar with a 0.7% egg-positive rate^27^. Thus, the present survey areas in Cambodia appeared to be one of the most highly endemic areas of *O. viverrini* infection ever reported.

There seem to be potential risk factors for the surveyed areas to appear as highly endemic areas of opisthorchiasis. One is the locality of the surveyed villages. These villages are located along the main stem or tributaries of the Mekong River. The 5 villages of Preah Vihear province locate just along the border between Cambodia and Lao PDR. In Lao PDR, *O. viverrini* infection has been known to be highly endemic, especially in Vientiane Municipality, Khammouane, Savannakhet, and Champasak provinces^18–22^. The 5 villages of Stung Treng province are located along the river and streams not far from the border area. Other risk factors include that most villagers are engaged in fisheries and are traditionally accustomed to eating raw or undercooked freshwater fish.

This study showed a significant age tendency in the prevalence of Ov/MIF infection. The prevalence was the lowest among children under 9 years, followed by the age groups 10–19 years and 20–29 years. It approached a peak at ages 30–39 years and then maintained the peak up to the age groups 40–49, 50–59, and ≥ 60 years. This tendency was like those reported previously for liver fluke infections in other countries; *O. viverrini* in Lao PDR and Thailand^28–30^, *O. felineus* in Russia^31^, and *C. sinensis* in South Korea^32^. Also, in Cambodia (Takeo province), almost the same age-prevalence pattern of *O. viverrini* infection was observed among the riverside people^4,6^.

Sex difference in the incidence of liver fluke infections is known to be variable according to locality and human behavior^31,32^, although helminth infections are generally known to be heavier in males than in females in mammalian hosts^33^. In South Korea, males revealed a higher incidence of *C. sinensis* infection than females, related to some social customs in males, i.e., frequent consumption of raw or undercooked freshwater fish with liquor in social gatherings^32^. Similar trends were observed in China, where *C. sinensis* is endemic^32^. Reversely, in *O. felineus* infection, the greatest incidence was noted among housewives, i.e., females, in Russia^31^. However, in Thailand, where *O. viverrini* is prevalent, male preponderance or no significant sex difference has been reported^20,31,34^. In Cambodia, a male preponderance was reported in Takeo province^6^. However, in the present study, no significant sex difference was noted. One of the reasons may be that the major source of infection in the surveyed areas is traditional fish dishes popularly consumed by the people regardless of gender, such as ‘*plea tre*’ (fish salad), ‘*plea tre chou*’ (sour fish salad), and ‘*ma’am*’ (fermented fish, kept for 2–5 days).

There was a positive correlation between the number of adult *O. viverrini* and/or *H. taichui* specimens recovered and the number of EPGs in 10 volunteers, as presented in Table 2. Our results agreed with a previous report from Thailand where 231 residents of a northeast community positive for *O. viverrini* eggs were treated with praziquantel, and adult flukes were collected^35^. The pre-treatment egg counts (EPG) showed a strong linear relationship with the number of worms recovered after the treatment. It was also of note that the fecundity of *O. viverrini* appeared to be related to the degree of parasite aggregation within the host body and negatively associated with the total worm burden in each host individual^35,36^. In the present study, the number of cases in whom worm recovery was performed was small (only 10), and the density-dependent reduction in worm fecundity was difficult to analyze.

It is noteworthy that *H. taichui* was found mix-infected with *O. viverrini* among the volunteers in Preah Vihear province. To the best of our knowledge, this is the first report of human *H. taichui* infection in Cambodia. This combination of mixed infections was previously found in other countries, including Lao PDR^19,21,22,37,38^ and Thailand^39,40^. One of the points that should be considered is the relative predominance of these fluke species. For example, in Saravane province, Lao PDR, only 68.4% (13/19) of the volunteers positive for Ov/MIF eggs in fecal examination expelled *O. viverrini* specimens after chemotherapy and purging, whereas 100% (19/19) of the volunteers did *H. taichui* flukes^41^. In addition, the total number of fluke specimens recovered was notably different; only 796 (n = 13) specimens of *O. viverrini* versus 409,738 (n = 19) specimens of *H. taichui*^41^. This^41^ and another study in Lao PDR^22^ support the finding that Saravane province is a highly endemic area of *H. taichui* infection with a low-grade infection with *O. viverrini*. Reversely, Vientiane Municipality was confirmed to be a highly endemic area of *O. viverrini* infection with a low-grade mixed infection with *H. taichui*^22^. On the other hand, Savannakhet province was an area with almost equal endemicity (prevalence and worm load) of *O. viverrini* and *H. taichui*^19^, whereas Khammouane province was an area with a slightly higher endemicity of *H. taichui* than *O. viverrini*^21^. Thus, the surveyed areas in our study, Preah Vihear and Stung Treng provinces, are regarded as an *O. viverrini* dominant area with a low-grade (low prevalence and low intensity) *H. taichui* infection.

In areas of mixed infections with *O. viverrini* and MIF (*H. taichui*, for example), molecular testing of the fecal eggs would be advantageous. Buathong et al. (2020)^42^ reported the usefulness of PCR and PCR-restriction fragment length polymorphism (PCR–RFLP) analysis targeting internal transcribed spacer 2 (ITS2) gene in differentially diagnosing *O. viverrini* and *H. taichui* infections in northern Thailand.

The average prevalence of *S. mekongi* appeared to be quite low, 0.8% in 10 villages surveyed, with a prevalence of 1.6% in Preah Vihear and 0.5% in Stung Treng province. These two provinces have been well known as the endemic areas of schistosomiasis mekongi, and long-time surveillance (1995–2010), as well as control efforts, have been implemented by the government^4^. The present findings seem to be the results of the governmental control activities against this trematode infection.

The eggs of *A. lumbricoides* were not detected in this study. This finding denotes that the national control program against soil-transmitted helminthiases in Cambodia by the mass drug administration (MDA) using mebendazole and/or albendazole twice a year for over 20 years^4^ has been highly successful. The prevalence of other soil-transmitted helminths appeared to be 12.2% for hookworms and 1.5% for *T. trichiura*; however, the prevalence of these parasites is also expected to decrease further so far as the national control program is continued. We regret that the prevalence of *S. stercoralis*, another soil-transmitted helminth species, could not be determined in this study because the Kato-Katz fecal examination method alone was applied. There were 6 people from whom female *E. vermicularis* adult nematodes were collected; among them, 5 people were egg-negative in Kato-Katz fecal examinations.

In conclusion, it has been confirmed for the first time in this study that the northernmost areas of Preah Vihear and Stung Treng provinces, Cambodia, bordering Lao PDR are highly endemic areas of *O. viverrini* infection, with a prevalence of 58.3 and 60.3% (59.8% in average), respectively. The adult flukes recovered from 10 volunteers in Kampong Sangkae village, Preah Vihear, were, in their majority, identified as *O. viverrini*. A substantial number of *H. taichui* adult specimens were also recovered from 7 of the 10 volunteers, together with a few or quite many specimens of adult nematodes (5 species) and cestodes (1 species). Further epidemiological studies of *O. viverrini* and *H. taichui* infections in humans should be performed in various localities along the Mekong River in Cambodia.

## Methods

### Ethical consideration and data processing

The National Ethics Committee for Health Research, Ministry of Health, Cambodia approved this study (approval no.: 099NECHR), and all the methods used were performed in accordance with the relevant guidelines and regulations. The CNM staff were informed of and oriented on the proposed project, explained about this project to the participants, and then the informed consent form was obtained from each participant for fecal examination and from each volunteer for adult worm recovery. The collected data were analyzed in Excel 2016 (Microsoft, Redmond, WA, USA) and were statistically evaluated using the chi-square test. The level of statistical significance was set at *P* < 0.05.

### Survey areas and participants

To determine the endemicity of *O. viverrini* and/or MIF among riparian people in the northernmost parts of Cambodia, a cross-sectional community-based survey was performed in April–May 2018 in 10 villages of Preah Vihear and Stung Treng province bordering Lao PDR (Fig. 1). These villages are located along the Mekong River; 5 villages in Preah Vihear are located along the mainstream, and the other 5 villages in Stung Treng are along a small tributary. These villages were selected in consideration of the previous results of fecal examinations in which small trematode eggs of about 28–30 μm in length (possibly the eggs of *O. viverrini* and/or MIF) were detected in a high proportion of the people examined (unpublished observations). The number of recruited people in this study was 432 males and 669 females, with an age range of 5–95 years. There were no special exclusion criteria for recruiting people for fecal examinations. Most residents in these villages were agricultural workers or fishermen along the Mekong River and popularly consumed traditional foods made of raw or undercooked freshwater fish.

### Fecal examinations

A total of 1101 fecal samples were collected from the people, one sample for each person, in the 10 selected villages of 2 provinces which were transported to the laboratory of the National Center for Parasitology and Malaria Control (CNM) in Phnom Penh in April–May 2018 within 24 h after collection. Fecal examinations were performed using the Kato-Katz thick smear method, which is useful for large-scale monitoring of helminthic infections, except for *Strongyloides stercoralis* larvae. One smear slide was prepared per person and examined under a light microscope in the CNM.

### Adult worm recovery and identification

Adult trematodes were recovered from 10 volunteers in Kampong Sangkae village, Preah Vihear Province, in December 2018. The reason for selecting this village was in consideration of the highest Ov/MIF prevalence among the 10 villages surveyed (see the “Results” Section). The inclusion criteria for volunteers were those showing > 240 EPG of Ov/MIF eggs, and the exclusion criteria included pregnant women, children younger than 15 years, and elderly people over 61 years. The adult worm recovery was performed in a regional health center within the village. The volunteers were provided with informed consent and were given orally 40 mg/kg single dose of praziquantel (Shinpoong Pharmaceutical Co., Seoul, Korea) plus 5–10 mg/kg pyrantel pamoate (Hangzhou Minsheng Pharm. Co., Hangzhou, China) followed by 40–50 g of magnesium sulfate for purging. The whole consecutive diarrheic stool passed 4–5 times after the medication was collected and washed several times with > 10 volumes of water and re-suspended in water. After 10 min, the clean upper layer was discarded, and the dark lower layer was examined under the naked eye or placed under a stereomicroscope (Olympus, Japan) and explored for worm parasites. The number of worms collected was counted for each helminth species. Some of the adult fluke specimens were fixed in 10% formalin under a coverslip pressure, stained with Semichon’s acetocarmine, dehydrated in a graded series of ethanol, cleared in xylene, and morphologically identified using a light microscope.

### Statistical tests

Statistical tests were performed using the R Statistical Software (v 4.1.2; R Core Team 2021, Vienna, Austria).

### Ethics approval

The study protocols were approved by the National Ethics Committee for Health Research, Ministry of Health, Cambodia (approval no.: 099NECHR). Informed consent was obtained from all the participants for fecal examinations and adult worm collection following chemotherapy.

## Data Availability

The datasets used and/or analyzed during the present study are available from the corresponding author.
